# 

**Published:** 1905-08

**Authors:** 


### CONTENTS.

ORIGINAL COMMUNICATIONS—

An Appeal for Prophylaxis of Venereal Diseases. By Louis C. Roughlin, M.D., Atlanta, Ga............. 289
Etiology and Pathology of Disease. By J. T. Roan, M.D., Quitman, Ga............................ 294

EDITORIAL NOTES AND COMMENTS—

The Yellow Fever.... ................................................. 302
The Portland Meeting................................................. 306

NEWS AND NOTES................................................. 311

CONTENTS CONTINUED................................................. X

